# Flow dynamics and pressure modulation in a patient-specific upper airway using a pulsating nasal jet

**DOI:** 10.1038/s41598-026-52238-8

**Published:** 2026-05-09

**Authors:** Muhammad Aseem, Elias Sundström, Liran Oren

**Affiliations:** 1 Depatment of Mechanical Engineering, University of Cincinnati, 598 Rhodes Hall, Cincinnati, Ohio USA; 2 Department of Engineering Mechanics, KTH Royal Institute of Technology, FLOW, 100 44 Stockholm, Sweden; 3 Department of Otolaryngology, Medical Science Building, University of Cincinnati, Cincinnati, Ohio USA

**Keywords:** Engineering, Health care, Medical research, Physics, Physiology

## Abstract

Pulsating airflow jets delivered via nasal cannula offer a promising, comfortable, non-invasive alternative to continuous positive airway pressure (CPAP) for treating obstructive sleep apnea (OSA). However, the fluid dynamic mechanisms by which pulsatile flow influences upper airway pressure remain poorly understood in anatomically realistic geometries. This study used large eddy simulations (LES) to examine pressure and flow characteristics of pulsating nasal jets within a patient-specific upper airway model. Two airflow conditions were simulated: (1) steady high-flow nasal cannula (HFNC) at 40 L/min and (2) pulsatile flow at 20 Hz with a 30% duty cycle, matched to the same mean flow rate. Each pulse generated a vortex ring that impinged on the nasal walls, creating localized high-pressure regions and asymmetric shear stress. Compared to steady flow, the pulsatile jet increased time-averaged pharyngeal pressure by up to 50%. Spectral analysis revealed that the 20 Hz pressure oscillations were primarily confined to the upper airway, with substantial attenuation through the pharyngeal–laryngeal region and negligible coherent oscillatory content by the trachea. These effects, shaped by jet-wall interactions in complex anatomy, diverge from classical vortex dynamics. Pulsatile nasal flow may offer a precise, geometry-responsive method for upper airway stabilization, suggesting a path toward a more tolerable, mask-free alternative to CPAP for OSA therapy.

## Introduction

Obstructive sleep apnea (OSA) is a common sleep-related breathing disorder characterized by recurrent collapse of the upper airway during sleep.^1^ This condition affects millions worldwide and is associated with serious health risks due to intermittent hypoxia and sleep fragmentation. The current gold-standard treatment, continuous positive airway pressure (CPAP), pneumatically splints the pharyngeal airway open.^2,3^ CPAP is efficacious when used consistently, but patients often cite the discomfort related to the tight-fitting mask required by current devices as the main reason for discontinuing their therapy.^4,5^ High-flow nasal cannula (HFNC) therapy has emerged as a less invasive alternative that can deliver modest positive pressure.^6,7^ In this therapy, continuous flow is injected into the nares via a nasal prong design, which is generally more comfortable than CPAP masks due to the absence of a required facial seal. However, HFNC typically cannot provide the pressure levels required for OSA therapy to prevent airway collapse.^8^ This limitation has motivated the exploration of novel non-invasive therapies that can enhance upper airway patency without the need for a face mask.

The fluid mechanics of continuous HFNC therapy have been investigated experimentally and computationally in prior studies, providing important insight into its pressure-generating capabilities and limitations. Experimental measurements and anatomically based computational fluid dynamics (CFD) simulations have shown that continuous HFNC produces only modest increases in intranasal and pharyngeal pressure, even at high flow rates. These studies indicate that much of the injected flow exits through the mouth or leaks around the cannula, and that a substantial portion of the pressure drop occurs across the nasal valve region, limiting effective pressure transmission to the pharynx. As a result, continuous HFNC typically generates pharyngeal pressures on the order of only a few centimeters of water, well below those required to reliably prevent upper airway collapse in OSA.^9–11^

Importantly, prior CFD investigations of HFNC have largely focused on steady or quasi-steady flow conditions, emphasizing mean pressure levels, resistance, and flow distribution within the nasal passages. While this work has established a mechanistic understanding of why continuous HFNC provides limited airway pressurization, it does not address how time-varying flow or unsteady jet dynamics might alter pressure transmission or local flow structures within the upper airway. In particular, the role of unsteady jet acceleration, vortex formation, and transient jet–wall interactions in modulating airway pressure has not been explored in anatomically realistic CFD models.

One promising approach is the use of pulsating airflow jets delivered via nasal cannulas.^12^ Unlike steady flow, a pulsatile jet produces dynamic pressure oscillations and coherent vortex structures that interact with the airway walls in unique ways. Prior observations showed that pulsating airflow can achieve therapeutic pharyngeal pressures equivalent to or exceeding those of CPAP in awake patients. This study demonstrated that pulsating nasal airflow delivered via a nasal cannula produced peak pharyngeal pressures up to ~20 cmH₂O. These values are significantly higher than those from HFNC (maximum of 5.1 cmH_2_O^6,7^), and match the typical pressure levels required for OSA therapy. However, translating this promising pulsatile-flow concept into a reliable therapy requires a deeper understanding of the underlying flow mechanisms in anatomically realistic airways.

It is well known that a starting jet or pulsed jets generate leading vortex rings that travel downstream. Upon impinging on a boundary, these rings can induce complex flow phenomena, including secondary vortices and localized pressure surges. Previous in vitro experiments^13–15^ involving vortex ring-wall interaction have shown that at sufficiently high Reynolds numbers, the primary ring expands, inducing boundary-layer separation that spawns secondary and even tertiary vortices. These interactions can cause rebound effects and eventual cascading flow breakdown. However, these studies were conducted in simplified geometries such as flat plates, inclined walls, or concave cavities, rather than anatomically realistic airways. For example, experiments with vortex rings impinging on inclined surfaces revealed asymmetric, helical vortex patterns.^14^ The portion of the ring hitting the wall first generates uneven vorticity and helix-like structures that migrate along the ring, away from the impact site. Similarly, vortex rings impacting confined concave cavities exhibit altered dynamics: increased vorticity at the cavity lip can disrupt the formation of classical secondary rings, and direct vortex impact on the cavity edge can even spawn additional vortex rings that travel in opposite directions.^13^ These prior works highlight how geometry and boundary conditions drastically influence vortex behavior. However, it remains unclear how such vortex–wall interaction principles translate to the upper airway, where the walls form a highly irregular, compliant passage.

Despite this promise, the mechanics of pulsatile jets in an anatomical airway remain incompletely understood. The upper airway has a complex geometry, characterized by narrow nasal passages, a sharp nasopharyngeal bend, and collapsible pharyngeal walls. How a train of vortex rings and jet bursts navigate this geometry, and how the resulting pressure field is distributed, is not obvious from the existing vortex ring literature. Using pulsating airflow as a therapeutic modality raises additional important questions: Do pulsatile jets predominantly create beneficial pressure elevations in the nose and pharynx? How quickly do the introduced vortex structures break down into turbulence in such a confined, winding airway? Are these large pressure oscillations transmitted into the lungs, or do they focus their effects more locally along the upper airways? The present study addresses these gaps by applying large-eddy simulations (LES) of pulsating nasal airflow in a patient-specific upper airway model. By integrating fluid dynamics insights from prior vortex ring experiments with state-of-the-art anatomical airflow modeling, we aim to elucidate the flow patterns and pressure oscillations generated by pulsating flow and to assess their potential to stabilize the airway for OSA therapy, thereby informing the design of next-generation mask-free OSA interventions.

## Methodology

### Upper airway model geometry

A patient-specific upper airway geometry was reconstructed from an anonymized maxillofacial CT scan of an adult subject with a clinical diagnosis of obstructive sleep apnea. The CT data were obtained from a prior study^16^, and provided sufficient spatial resolution for the present computational analysis. The present work is intended as a patient-specific, mechanistic case study, and no prospective anatomical selection criteria were applied. All methods were carried out in accordance with relevant guidelines and regulations. The study protocol was reviewed and approved by the University of Cincinnati Institutional Review Board, which determined that the use of fully de-identified CT images did not require informed consent.

**Fig. 1:**
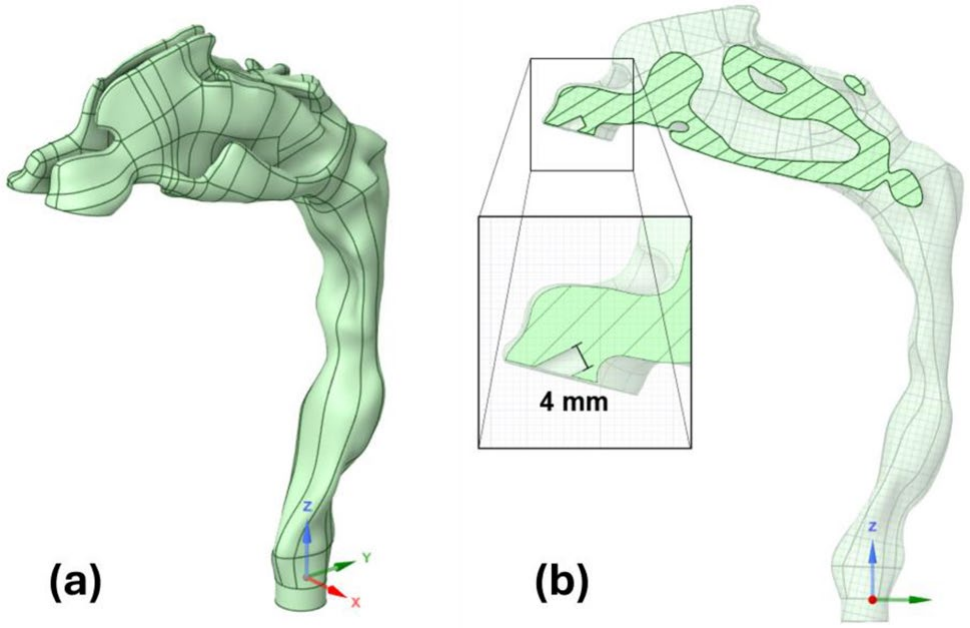
Patient-specific upper airway geometry reconstructed from CT imaging. (**a**) Isometric view showing the full computational domain from the nares (inlet) to the tracheal outlet below the larynx. **b**) Sagittal cross-section showing nasal prong insertion at ~45° relative to the hard palate. A zoomed inset highlights the 4 mm inner diameter of the nasal prongs used to model a high-flow nasal cannula interface.

Each CT slice was 0.63 mm thick with an in-plane resolution of 0.43 mm/pixel. The airway lumen was segmented from the CT images using the open-source software 3D Slicer, employing intensity-based thresholding followed by manual slice-by-slice refinement to ensure continuity of the airway lumen.^17^ The resulting 3D model (STL format) was refined in MeshLab^18^ and subsequently imported into ANSYS SpaceClaim to create a smooth solid CAD model. Only light smoothing was performed to remove pixelation artifacts, with no changes to the overall airway dimensions or crucial passage areas. The model domain extends from the nares (nostril openings) at the inlet to the tracheal section just below the larynx (Fig. 1a). The paranasal sinuses were excluded from the computational domain because they are weakly ventilated during inspiration and have minimal influence on bulk nasal airflow, jet propagation, and pressure transmission under the flow conditions considered here. The model also included simulation of nasal prongs (4 mm inner diameter) that were inserted ~6 mm into each nostril at ~45° relative to the hard palate (Fig. 1b), approximately along the centerline to maximize jet penetration before impingement on the nasal wall. This geometric setup was designed to mimic a high-flow nasal cannula interface.

The selected anatomy exhibits asymmetry between the left and right nasal passages, which is common in the adult population (Table 1). Nasal septal deviation and associated asymmetry are highly prevalent and are often present even in individuals without clinically significant nasal obstruction.^19^ The present work is a mechanistic, patient-specific case study of a subject with an OSA diagnosis and notable left-right nasal asymmetry. The observed findings should be interpreted within this specific clinical and anatomical context rather than as representative of canonical nasal airflow or population-level patterns. However, the asymmetry provides a realistic test of how pulsatile jet dynamics interact with anatomically non-ideal airways, which are common in clinical practice.

**Table 1:** Cross-sectional area at each measurement plane along the patient-specific upper airway model (see Fig. 3b for locations). Planes A1–A4 report separate left and right nasal cavity areas.

**Plane**	**Region**	**Left Area (mm**^**2**^**)**	**Right Area (mm**^**2**^**)**
A1	Nasal cavity	93	131
A2	Nasal cavity	148	131
A3	Nasal cavity	219	209
A4	Nasal cavity	177	197
A5	Nasal cavity	425	
A6	Oropharynx	527	
A7	Oropharynx	300	
A8	Oropharynx	121	
A9	Pharynx	99	
A10	Pharynx	278	
A11	Pharynx	123	
A12	Larynx	131	
A13	Trachea	127	

Detailed anatomical descriptors such as minimum cross-sectional area and unilateral nasal resistance were not the focus of this mechanistic, patient-specific study and are therefore not reported. The present work focuses on pressure modulation and spatial pressure distribution under matched mean flow conditions rather than estimating airway resistance, which depends on airway segment definition and operating conditions and would require a systematic pressure–flow analysis beyond the scope of this study.

The airway volume was discretized using an unstructured tetrahedral mesh generated in ANSYS Meshing. We applied fine mesh refinement in the nasal vestibule and valve regions to capture steep velocity gradients. Five inflation layers (with a growth rate of 1.2) were added at the walls to resolve the boundary layer. The anterior nasal cavity, with its intricate geometry, was meshed at roughly twice the base resolution to capture the complex flow patterns within it better. The final mesh contained approximately 9.5 million elements.

### Boundary conditions

**Fig. 2:**
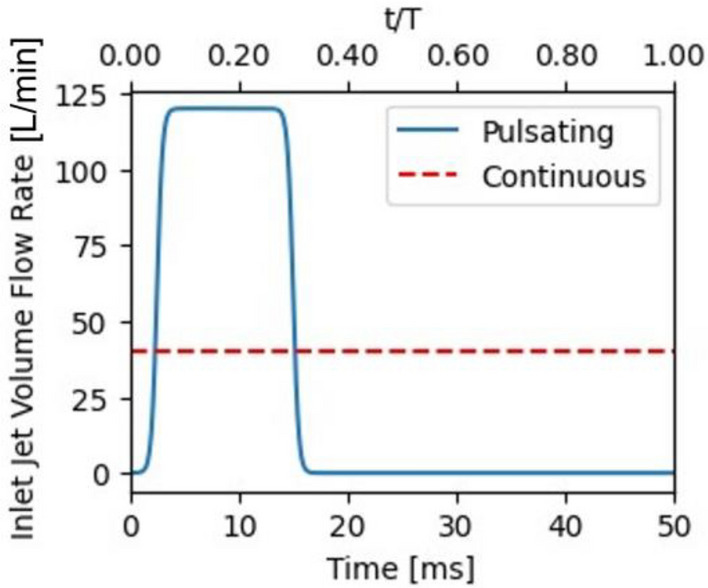
Inlet flow rate waveforms for the nasal cannula. The continuous case (dashed red line) is a steady 40 L/min flow. The pulsatile case (solid blue line) delivers the same 40 L/min mean flow via 20 Hz pulses with a 30% duty cycle (peaks of 120 L/min during each pulse).

Boundary conditions were designed to replicate a strong inspiratory effort combined with supplemental flow from nasal cannula prongs. A baseline inhalation flow of 30 L/min was applied uniformly across the nares inlet plane, representing a normal-to-heavy peak inspiratory flow.^20^ Superimposed on this, we modeled two cases of uniformly-distributed nasal cannula airflow: (1) a continuous steady jet of 40 L/min, and (2) a pulsatile jet with a mean flow of 40 L/min delivered as 20 Hz bursts at 30% duty cycle (these parameters were selected following prior work demonstrating effective pressure generation at high-frequency oscillations^12^, and reflects a feasible operating range for pulsatile flow devices. In the pulsatile case, each “on” phase lasted 15 ms with a peak flow of 120 L/min during that interval, such that the time-averaged flow matched 40 L/min over a full cycle. The steady inspiratory baseline was chosen to decouple pulsatile jet dynamics from lower-frequency respiratory transients and to enable controlled comparison between continuous and pulsatile injection cases. Figure 2 illustrates the mass flow rate waveforms for the continuous and pulsatile injection. Boundary smoothing was applied with the pulsatile injection to model the waveform transitions. All walls were treated as no-slip boundaries, and a constant gauge pressure of 0 Pa (i.e., atmospheric) was imposed at the distal (tracheal) outlet.

### Simulation setup

All simulations were performed using ANSYS Fluent. Each case (continuous and pulsatile) was simulated for a physical duration of 0.1 sec. For the continuous 40 L/min flow, this duration was sufficient to reach a quasi-steady state, as monitored flow variables became nearly constant in time. The pulsatile case covered two full pulse cycles (each 50 ms long) to observe periodic behavior.

An implicit unsteady solver with a bounded second-order time integration scheme was used to accurately capture transient dynamics.^21^ Large-eddy simulation (LES) was employed with the Wall-Adapting Local Eddy-viscosity (WALE) subgrid-scale model to resolve the dominant unsteady flow structures while accounting for near-wall effects. The choice of LES over steady or unsteady RANS was motivated by the need to resolve transient vortex formation, impingement, and breakdown events that are central to the pressure modulation mechanisms investigated here. RANS models, by design, model all turbulent fluctuations and cannot capture the formation and evolution of coherent vortex rings, their interaction with airway walls, or the resulting transient pressure surges. Unsteady RANS (URANS) can resolve low-frequency unsteadiness but typically fails to capture the rapid spectral broadening and vortex breakdown that occur upon wall impingement in confined geometries. The pulsatile jet produces flow features, including coherent vortex rings, transient stagnation pressure peaks, and rapid transition to broadband turbulence, that lie in the resolved-scale regime of LES but would be entirely modeled (and thus absent from the solution) in a RANS framework. Because these features are the primary subject of this study, LES is the minimum-fidelity approach capable of addressing the research questions posed.

The time step was fixed at 2 × 10⁻⁶ s, which maintained a convective Courant number below unity in the regions of highest velocity gradients, particularly near the nasal prong exits and jet impingement zones. A formal time-step sensitivity study was not performed due to computational cost; instead, temporal adequacy was assessed using Courant number control in the jet region and independent spectral diagnostics of the resolved velocity fluctuations, as described below.

The temporal resolution was selected based on the dominant unsteady flow mechanisms of interest rather than on wall-unit time scales associated with fully developed internal turbulence. In this problem, the primary unsteady dynamics arise from the imposed high-frequency pulsatile jet, including pulse onset, jet acceleration, vortex formation, and subsequent vortex–wall interactions within the nasal cavity. These processes are strongly forced and occur on time scales set by the pulse period and the advection of large, energy-containing vortical structures, rather than by equilibrium near-wall turbulence production.

The chosen time step resolves the pulse “on” phase with multiple time steps and captures the formation and downstream convection of the jet-generated vortices without excessive temporal filtering. This ensures adequate temporal resolution of the large-scale flow structures that dominate pressure generation in the upper airway. While wall-unit time scales are commonly reported in canonical wall-resolved LES of fully developed internal flows, they are less relevant here because pressure modulation is governed primarily by large-scale jet–wall interactions and flow separation.

**Fig. 3:**
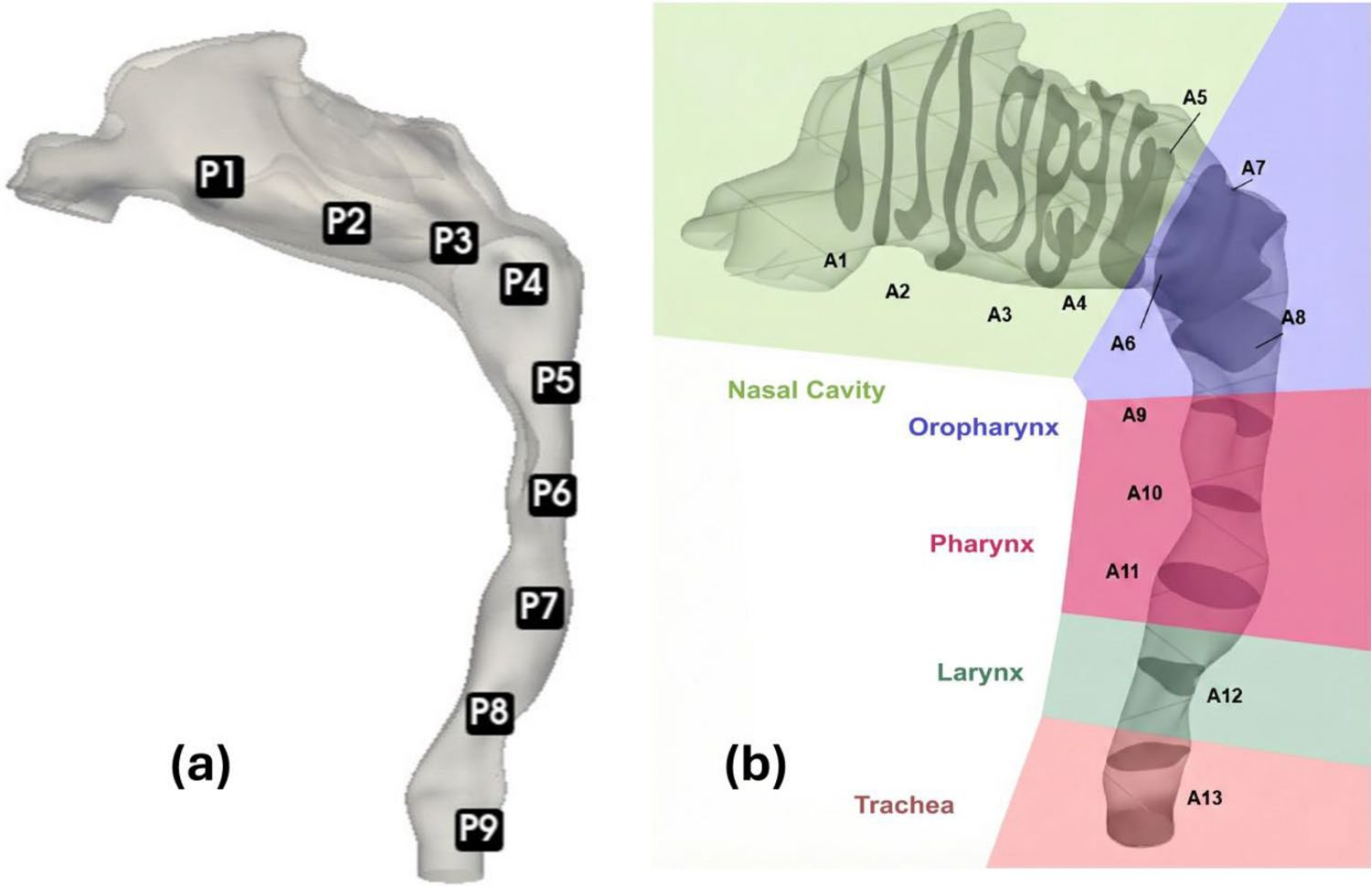
Locations of monitoring points (**a**) and cross-sectional data planes (**b**) defined along the airway model for analysis. A total of 9 points (centerline locations) and 13 perpendicular planes span the nasal cavity (P1-P3, A1-A5), oropharynx (P4, A7-A8), pharynx (P5-P7, A9-A11), larynx (P8, A12), and trachea (P9, A13).

Temporal adequacy was further assessed through spectral analysis of velocity fluctuations (Section "Energy spectral analysis"), which demonstrates a resolved inertial subrange and indicates that the selected time step does not unduly damp the resolved turbulent scales. Flow field data (velocities and pressures) were recorded every 0.005 s at specified monitoring points and cross-sectional planes throughout the domain (Fig. 3). Pressure–velocity coupling was handled using the PISO algorithm, chosen for its efficiency and stability in resolving fast transients.^22^ The computations were executed on the Ohio Supercomputer Center using 140 CPU cores distributed across five nodes.

All LES simulations were initialized from a converged steady-state RANS solution to minimize initial transients. The continuous-flow case was advanced until monitored global pressure and velocity signals reached quasi–steady behavior prior to data extraction. For the pulsatile case, the simulation was advanced over multiple pulse cycles until repeatable, cycle-to-cycle behavior of monitored pressures and velocities was observed, after which statistics were phase-averaged over a complete pulse period.

## Model validation and verification

### Comparison with experiment

**Fig 4:**
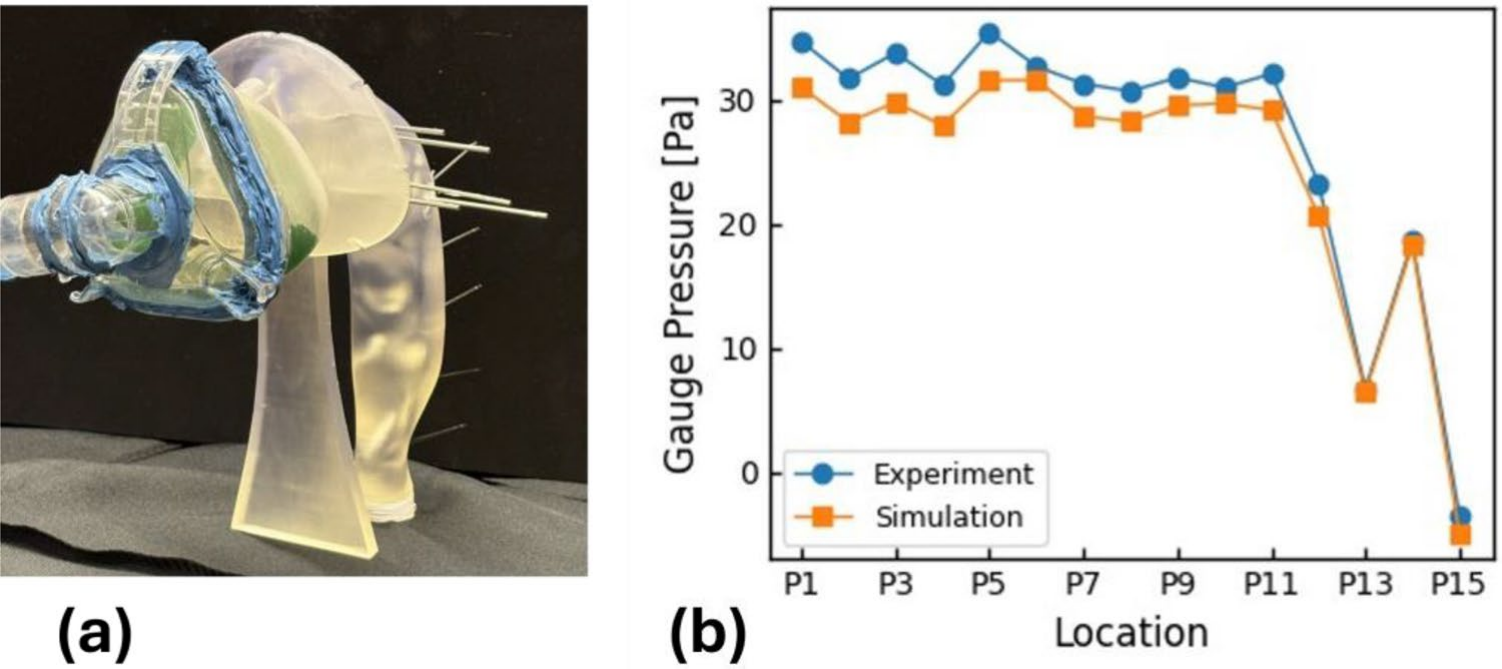
Experimental setup and pressure validation. **a**) 3D-printed airway model with wall pressure ports (P1–P9) for pressure measurements. **b**) Comparison of measured transmural pressure (points) vs. CFD-predicted pressure (line) along the airway for a steady 30 L/min inspiratory flow.

To validate the CFD model, we conducted an experimental measurement of airway pressures using a life-size replica of the same anatomy. The patient-specific airway was 3D-printed (stereolithography) and instrumented with 15 small pressure taps (1.6 mm diameter) along the airway wall (Fig. 4a). The pressure port locations were located on the walls at the same levels as in the simulations. Each port was connected to a pressure transducer (Honeywell FPG, 0–5 inH₂O) and sampled at 1000 Hz for 1 s using an NI-9234 data acquisition system. In the experimental validation, airflow was delivered through a facial interface that preserved the external nasal geometry, whereas the computational model prescribes inlet conditions at the nostril openings and focuses on reproducing intraluminal pressure behavior.

Replicating the exact combined flow condition of the simulation (simultaneous 30 L/min inspiration plus pulsation) was not feasible with our benchtop setup. Therefore, we performed the experiment under the baseline inhalation flow only. A constant 30 L/min airflow was delivered through a nasal mask attached to the model’s nares (Fig. 4a), matching the simulation’s inspiratory boundary condition. Figure 4b compares the measured transmural pressure (inside minus ambient) at each port with the pressure predicted by the CFD for the same 30 L/min steady inflow.

The simulation and experiment show good agreement in pressure distribution (Fig. 4b). The mean gauge pressure measured on the wall is compared with the predicted value at the monitoring point. The steady 30 L/min flow generated a slight positive pressure in the nasal cavities and nasopharynx, which then decreased through the pharyngeal-tracheal region. Both the measured and simulated pressure profiles exhibit a small pressure recovery at port P8, corresponding to a local airway expansion in the pharynx that converts kinetic energy back into pressure. The largest discrepancy was approximately 11.9% at one of the nasal cavity ports (P2), but overall, the absolute mean error between simulation and experiment was only 4.63%. This level of accuracy gives confidence that the CFD model can reliably capture pressure behavior in the upper airway under the given flow conditions.

### Grid sensitivity

A mesh sensitivity study was conducted using four progressively refined unstructured grids containing approximately 6M, 8M, 9.5M, and 12M cells. The predicted pressure fields were compared across these grids. We found that increasing the mesh from 9.5M to 12M cells changed the pressures by less than 2%, time-averaged over the length of the pulse and area-averaged across each cross-section. Additionally, no discernible changes were observed in the evolution of the vortex ring structures or wall shear stress distributions between these two meshes. Based on this convergence of both integral and structural flow metrics, the 9.5M-cell mesh was deemed sufficient for all subsequent simulations.

### Energy spectral analysis

**Fig. 5:**
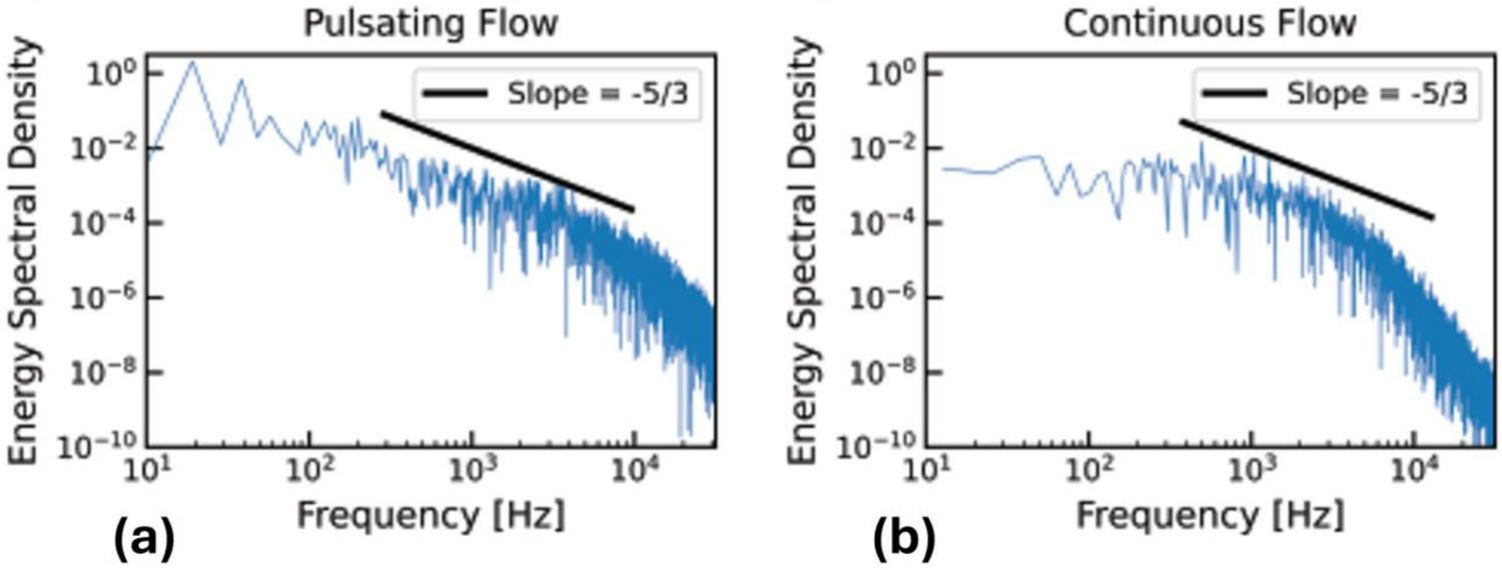
Energy spectral density of velocity fluctuations at a nasopharyngeal monitoring point (P4). Over a limited mid-frequency range, the spectrum exhibits an approximately –5/3 slope (solid black line), which is commonly associated with inertial-range scaling in turbulent flows. In the present context, this behavior does not imply fully developed turbulence, but rather indicates that the LES resolves a range of dynamically relevant unsteady scales without excessive numerical dissipation. **a**) Pulsating flow. **b**) Continuous flow.

As an additional verification of the LES solution quality, we examined the energy spectral density of velocity fluctuations at a representative point in the flow. Specifically, the velocity signal was sampled at a monitoring point located in the nasopharynx (P4). The spectrum was obtained by performing a Fast Fourier Transform on a time series of velocity fluctuations sampled at 500 kHz over a duration of 10.8 ms, after removal of the mean velocity component. The resulting spectrum (Fig. 5) exhibits an approximately –5/3 slope over a limited mid-frequency range spanning roughly one decade. This behavior is commonly associated with inertial-range scaling in turbulent flows and, in the present context, indicates that the selected spatial and temporal resolution resolves a range of dynamically relevant unsteady scales without excessive numerical dissipation. This analysis is intended as a diagnostic of LES resolution quality rather than as evidence of fully developed turbulence or an extended inertial subrange. Figure 5a corresponds to pulsatile flow and Fig. 5b to continuous flow.

## Results

### Opening vortex formation at pulse onset

**Fig. 6:**
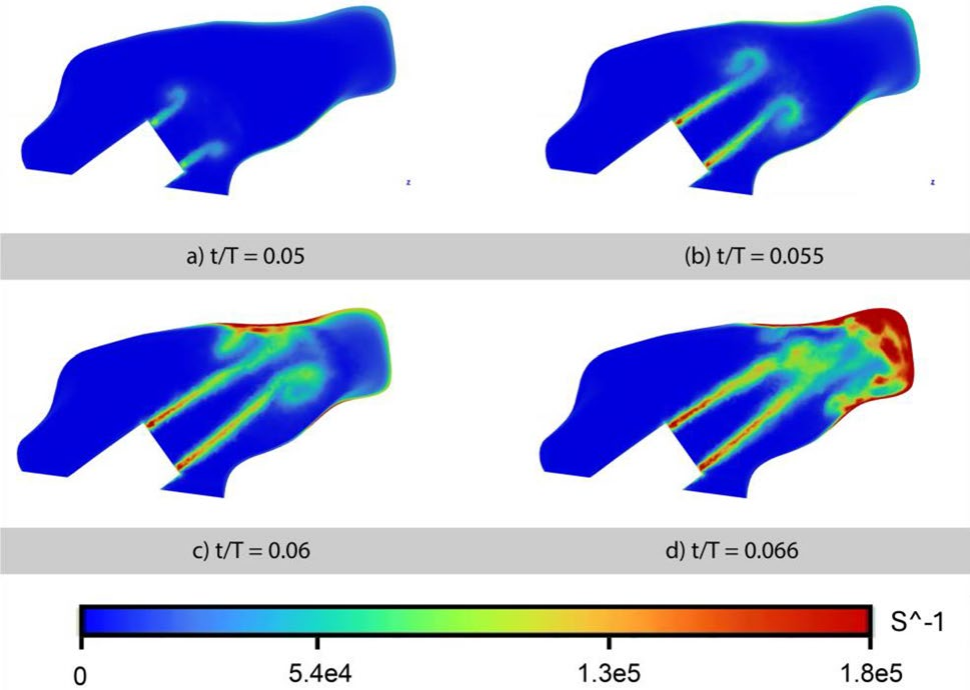
Vorticity contour snapshots at the onset of a pulsatile jet flow, illustrating the formation and advection of an “opening vortex” ring just downstream of the nasal prong. Times shown correspond to (**a**) 0.050, (**b**) 0.055, (**c**) 0.060, and (**d**) 0.066 of the pulse cycle (t/T). The vortex forms near the prong exit and travels into the nasal cavity, temporarily elevating local pressure before impinging on the nasal wall. An animation showing the full pulsatile cycle is provided as Supplementary Video 1.

At the start of each pulsation cycle, the sudden jet of airflow generates a distinctive vortex structure within the nasal cavity. Fig. 6 shows vorticity contours in a sagittal slice through the nasal passage during the initial milliseconds of the pulse. By time $t/T=0.050$ (where $T$ is the 50 ms pulse period), a coherent vortex ring begins to form just downstream of the nasal prong exit. This structure is characterized by a concentrated region of high vorticity and a well-defined core, corresponding to a local increase in static pressure resulting from induced pressure and local momentum deceleration. As the pulse continues to $t/T=0.055$, the vortex detaches from the jet and convects downstream along the airway. By $t/T=0.060$, the vortex is fully developed and moving further downstream. In our model, the vortex eventually impinges on the nasal vestibule wall by around $t/T=0.066$ (Fig. 6d). While the exact timing and location of vortex impingement would likely vary with different prong insertion angles or patient anatomies, the general phenomenon of a pulse-generated vortex is expected to occur in pulsatile nasal flows. This “opening vortex” is analogous to the starting vortex ring produced by a pulsed free jet, which typically generates an initial pressure surge upon formation. However, in the confined nasal cavity, this vortex quickly impinges on the walls, altering its development as described. The full temporal evolution of the jet and vortex structures over a complete pulsation cycle is shown in Supplementary Video 1.

### Spatial velocity distribution during mid-on-pulse

**Fig. 7:**
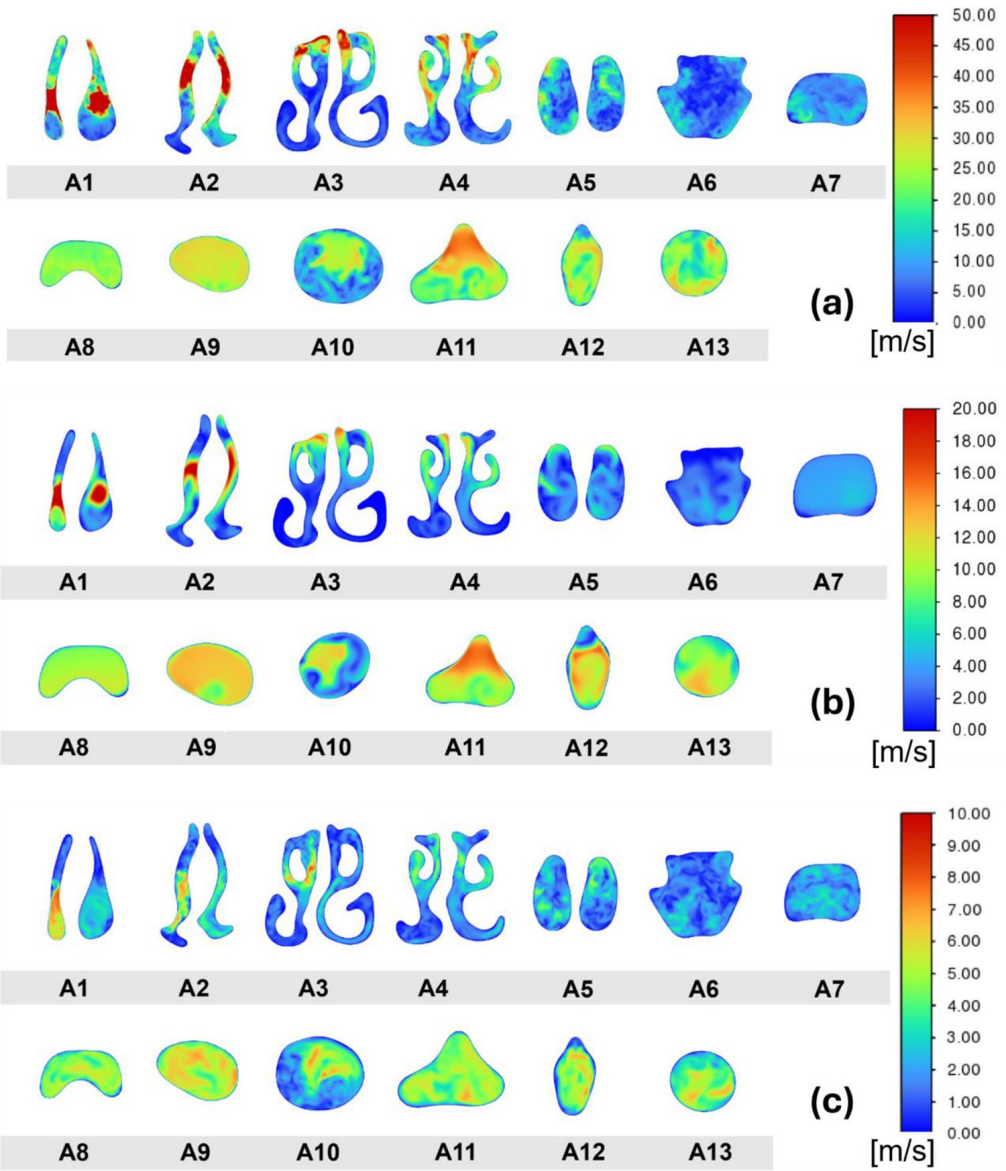
Cross-sectional velocity magnitude contours under different conditions. **a**) Pulsatile flow at mid-on-pulse (t/T = 0.15). **b**) Continuous flow at 40 L/min. c) Pulsatile flow at mid-off-pulse (t/T = 0.65). The pulsatile case exhibits similar flow patterns to the continuous case, but with much higher peak velocities during the active pulse and markedly lower velocities during the no-flow interval compared to the steady case. The apparent disturbance near the superior meatus reflects localized interaction of the pulsatile jet with patient-specific anatomy during peak injection phases and does not imply sustained high flow or persistent redirection of airflow into the olfactory region.

At mid-pulse ($t/T=0.15$), the airflow velocity field shows complex spatial patterns through the airway. Figure 7a presents velocity magnitude contours on a series of cross-sectional slices (A1–A13) spanning from the nasal vestibule to the trachea. In the anterior nasal cavity (slices A1–A3), the pulsatile jet creates narrow regions of very high velocity adjacent to regions of low velocity, reflecting the jet’s interaction with the nasal walls and the previously described opening vortex. At slices A1 and A2, these high velocity magnitude regions trace the jet trailing the vortex, and at A3 the vortex impinges on the superior nasal cavity wall, causing a localized peak in velocity. As the airflow progresses posteriorly, it decelerates upon entering the wider nasopharynx (A5) and slows further in the oropharynx (A6–A8) due to increased cross-sectional area. After passing through the constricted laryngeal region (around slice A12), the flow re-accelerates slightly into a jet-like profile in the pharynx (A10–A11) but then slows again in the trachea (A13). The sudden expansion beyond the larynx leads to flow separation from the wall and a recirculation zone forming in the upper trachea, as indicated by reversed or low-velocity flow near the airway walls in those downstream slices.

The transient flow disturbances observed near the superior meatus in Fig. 7 occur primarily during the peak phase of the pulsatile injection and reflect local jet–wall interactions in a highly asymmetric nasal geometry. These disturbances are not representative of sustained bulk airflow into the olfactory region, which remains weakly ventilated under normal inspiratory conditions. The pronounced skew of flow toward the superior meatus in this subject could be attributed to anatomical septal deviation. However, external facial geometry was not included in the model, but the good match of intraluminal pressures with experiment suggests that this simplification did not significantly distort the internal flow distribution. The present analysis focuses on pressure modulation and jet dynamics within the main nasal passages, and does not imply chronic alteration of airflow to olfactory structures. Assessment of long-term olfactory exposure or mucosal transport is beyond the scope of this study.

When compared to the continuous flow case (Fig. 7b), the spatial distribution of velocities is qualitatively similar – the high-speed regions occur in roughly the same locations along the airway for a given mean flow rate. However, the magnitudes differ markedly. The continuous 40 L/min jet produces much lower peak velocities than the pulsatile jet. In other words, with both cases delivering the same mean flow, the pulsating injection results in much higher instantaneous velocities during the pulse than the steady flow does at any time.

### Spatial velocity distribution during mid-off-pulse phase

During the pulsatile flow’s off phase (mid-shut-off, $t/T=0.65$), the flow distribution changes significantly in the nasal region. With no jet being injected from the prongs during this phase, the only airflow arises from the baseline inspiratory flow of 30 L/min entering through the nares. Thus, velocity magnitudes throughout the nasal cavity are substantially lower than during the pulse-on phase (Fig. 7a vs. Fig. 7c). In fact, the pattern in Fig. 7c represents a normal peak inspiratory flow distribution without any (pulsatile or continuous) flow augmentation. Downstream of the nasal cavity, the contours in the oropharynx, pharynx, and trachea remain qualitatively similar in shape to those observed during mid-pulse. The airflow still accelerates through narrower sections and decelerates in expansions, but all velocity values are much smaller without the pulsatile boost. Essentially, the pulse-off phase resembles a low-speed version of the flow, confirming that the added jet is responsible for the high-velocity peaks observed during the on-phase.

### Pressure distribution

**Fig. 8:**
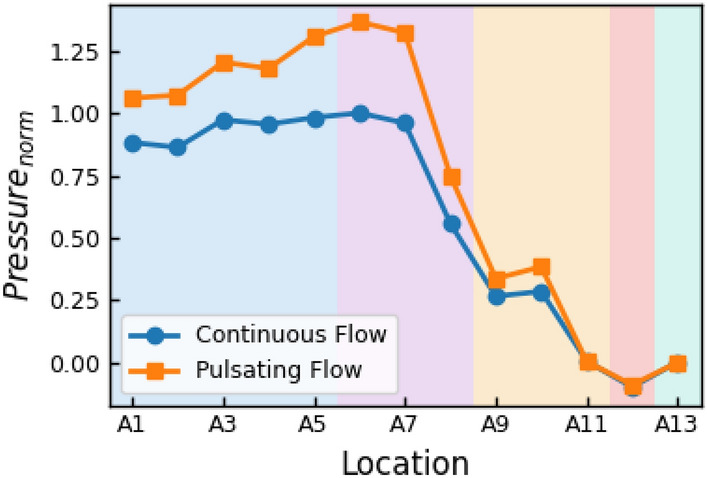
Normalized mean pressure distribution along the airway for pulsatile versus continuous flow. Pressures are normalized by the peak mean pressure of the continuous-flow HFNC case (~1.5 cmH₂O), which is consistent with previously reported HFNC pressure levels in CFD and experimental studies.^10^ This normalization highlights relative pressure amplification due to pulsation and does not represent absolute resistance changes.

A primary goal of introducing pulsating jets is to further increase the mean airway pressure beyond the elevated levels already achieved with HFNC. The mean pressure distribution along the airway model was calculated for each of the data planes (Fig. 8). Because the mean volumetric flow rate is matched between the pulsatile and continuous cases, the pressure differences shown reflect differences in flow modality and unsteady jet dynamics rather than changes in bulk nasal resistance. To emphasize the difference between cases, all pressure values were normalized by the maximum pressure value calculated in the continuous flow case (predicted to occur in the oropharynx). In the pulsatile case, “mean” refers to the time-averaged pressure over a full cycle, allowing for direct comparison with the steady case.

To provide external context for the measured and predicted pressure levels under continuous high-flow conditions, we compared our results with previously published experimental studies of HFNC. Moore et al. reported pharyngeal and tracheal pressures on the order of 1.5–2.0 cmH₂O for adult airway replicas under continuous HFNC at comparable flow rates using bench-top experiments.^10^ The numerically predicted peak continuous-flow pressures (~1.5 cmH₂O) in the present study are in close agreement with these experimental values, supporting the realism of the continuous-flow baseline used for normalization and comparison.

Both the continuous and pulsatile flows exhibit a notable pressure drop from the nasal cavity into the oropharynx. This can be explained by the flow convergence and acceleration in that region: the two streams from the left and right nasal passages combine into one nasopharyngeal airway, whose cross-sectional area is less than the sum of the two nasal cavities. By the continuity principle, the airflow must speed up as it passes through this bottleneck, and according to Bernoulli’s equation, a higher velocity corresponds to a lower static pressure. Additionally, the airway path makes a turn (approximately 90°) as it transitions from the horizontal level of the nasal cavity into the vertical level of the oropharynx. This change in flow direction causes a loss of momentum, further contributing to the pressure drop between the nasopharynx and oropharynx (specifically, between A7 and A8). The pressure continues to decline slightly through the pharynx, then beyond the pharyngeal region, it levels off. From the open larynx into the trachea, the cross-sectional area remains fairly constant, and thus the pressure remains relatively uniform. This overall pressure distribution pattern underscores the dominant role of airway geometry in shaping where pressure losses occur.

The present study focuses on a single clinically relevant high-flow condition to isolate the effects of pulsatile versus continuous injection, and a systematic flow-rate sweep is beyond the scope of this mechanistic case study.

### Wall shear stress

**Fig. 9:**
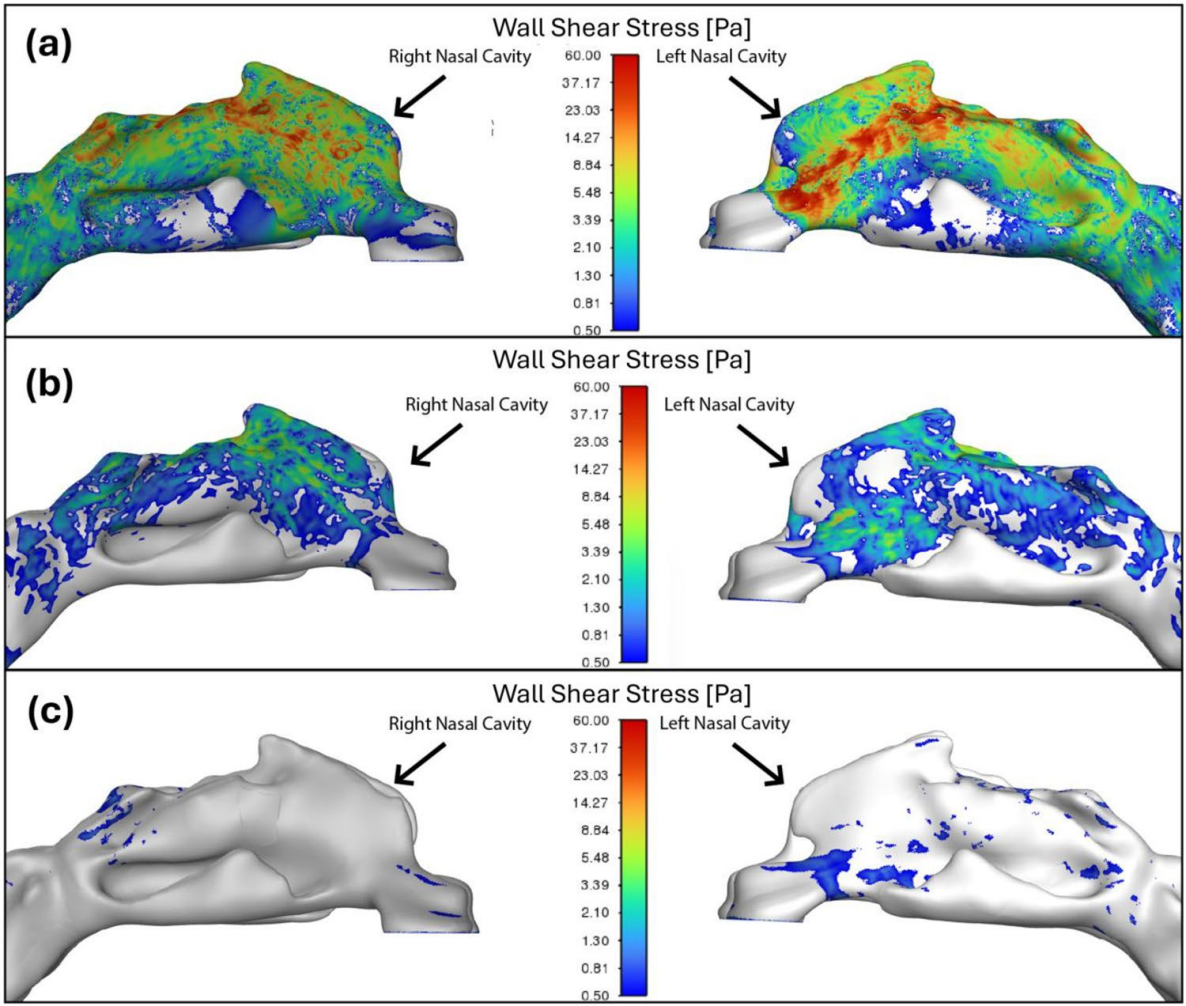
Wall shear stress (WSS) contours on the airway walls. **a**) Pulsatile flow during the on phase of the injection cycle. **b**) Continuous flow at the same mean volumetric flow rate. **c**) Pulsatile flow during the off phase, which closely resembles peak inspiratory flow without jet injection. Phases shown are the same as in Fig. 7. Elevated WSS during the pulsatile on phase reflects localized, transient jet–wall interaction and does not represent baseline physiological shear levels. The left nasal cavity (arrow) experiences higher localized shear during the pulsatile on phase due to patient-specific narrowing, whereas shear levels during the off phase fall within ranges commonly reported for normal inspiratory nasal airflow.

Wall shear stress (WSS) quantifies the drag force of the airflow acting on airway surface and is a key determinant of mucosal stimulation, potential tissue irritation, and patient comfort. The WSS is mapped throughout the airway for both continuous and pulsatile flows (Fig. 9). A striking feature is the left–right asymmetry: due to the patient-specific anatomy in this case, the left nasal passage experiences much higher shear stress than the right side. In this individual, the left nasal cavity’s airflow is directed toward the olfactory region, causing elevated shear in that area, whereas the right cavity has a more open path that produces relatively little shear on the walls. Beyond the nasal cavity, WSS levels drop off dramatically; in the pharynx, larynx, and trachea, wall shear stress is less than 1% of the maximum nasal wall shear stress during continuous flow and pulsatile on phase conditions, because the airflow core is largely separated from the walls. This left–right disparity in wall impact is reminiscent of a vortex ring striking an inclined surface, where the near-wall side of the ring generates stronger vorticity and more complex flow than the far side.

The elevated WSS observed during the pulsatile on phase are a direct consequence of the imposed high-velocity jet and are intentionally supra-physiological. These peak shear values, while above normal inspiratory levels, occur over very small areas and only during the brief pulse-on period. These values should be interpreted as transient, localized loading rather than as representative of resting nasal airflow. During the off phase of the pulsatile cycle, which closely resembles peak inspiration in the absence of jet injection, wall shear stress levels fall within ranges commonly reported for physiological nasal airflow. The strong left right asymmetry reflects patient-specific airway narrowing and highlights how anatomy modulates local shear amplification under pulsatile forcing.

When comparing flow modes, the pulsatile jet’s impact on shear stress is evident. Under continuous flow (Fig. 9b), the maximum shear stress on the left nasal wall is moderate. But during the pulsatile flow’s active phase (Fig. 9a), the peak WSS on that same wall is roughly three times higher. The pulsation drives a stronger jet against the nasal wall on one side, amplifying shear stress there. During the pulsatile off-phase (Fig. 9c), the shear pattern resembles the continuous case (since only the base inspiratory flow is present). The right nasal cavity remains low-shear in all scenarios.

### Spectral analysis

Whereas Section "Pressure distribution" examines time-averaged pressure levels along the airway, the analysis below focuses on the spectral content of pressure fluctuations about the mean.

**Fig. 10:**
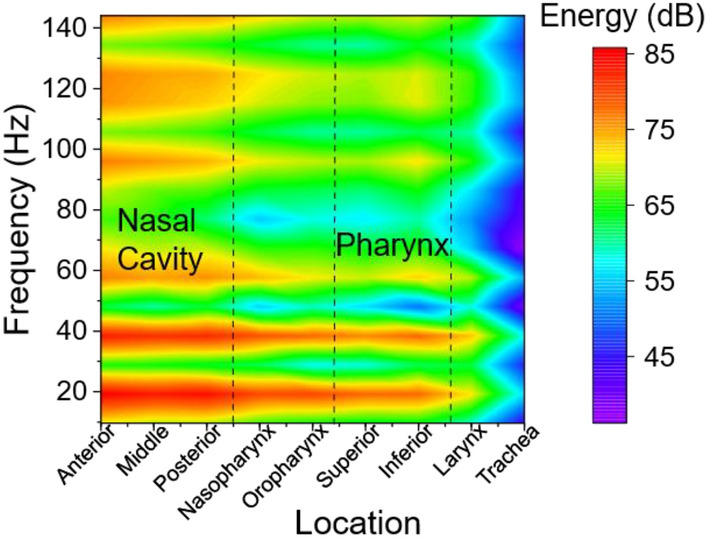
Pressure spectral density distribution along the airway model. The 20 Hz fundamental frequency corresponding to the injected pulse rate remains clearly identifiable along the airway, although its relative amplitude decreases downstream as energy is redistributed to higher frequencies. This behavior indicates progressive spectral broadening of the pressure signal due to unsteady jet interactions and geometric complexity, rather than loss of the imposed oscillatory forcing.

The frequency content of pressure fluctuations at 13 cross-sectional planes along the model (Fig. 10) was analyzed to understand how the pulsation propagates and dissipates through the airway. A spatial contour plot, similar to a spectrogram, was created from the pressure spectra computed at each plane using FFT on time-resolved pressure signals. At the nasal inlet, the spectrum exhibits a dominant peak at 20 Hz, which is the driving frequency of the pulsating jet. This 20 Hz component gradually diminishes in strength downstream. By the time the flow reaches the end of the oropharynx, the amplitude of the 20 Hz peak is significantly reduced. Below the larynx, the 20 Hz signal is barely discernible above the background. Instead, higher-frequency components become prominent in these distal regions, indicating a transition from coherent pulsation to broadband turbulent fluctuations.

Although the magnitude and spatial distribution of spectral energy are influenced by the pronounced left-right asymmetry of the airway, the injected 20 Hz fundamental frequency remains clearly identifiable along much of the airway, indicating a robust response to pulsatile forcing that is not specific to a single nasal cavity.

This trend suggests that the coherent pulsation imposed by the device is largely absorbed or broken down by the complex upper airway. While the initial pulse injects energy at a discrete frequency of 20 Hz, the flow encounters the complex, curved geometry and undergoes progressive spectral broadening due to unsteady jet–wall interactions; much of that energy is redistributed across a wider spectrum, including smaller-scale, higher-frequency eddies. The oropharynx appears to be a key region where the organized pulsation loses coherence, likely due to flow separation and vortex dissipation there. Consequently, the 20 Hz oscillation is essentially absorbed in the upper airway, with negligible transmission to the lower airway. This rapid breakdown of the coherent pulsation is consistent with prior observations of vortex rings dissipating into turbulence after wall impact, and it underscores that the pulsatile energy remains localized to the target region (nasal–pharyngeal airway).

## Discussion

### Comparison to prior vortex ring studies

The current results show that each pulse generates a pronounced vortex ring (an “opening vortex”) in the nasal cavity, which subsequently impinges on the airway walls. The vortex structures observed here are consistent with prior studies of pulsed jets and impinging flows in idealized geometries.^23^ However, their interaction with the highly nonuniform nasal airway produces anatomy-dependent pressure responses that are not captured in simplified models. In the simulations, the nascent vortex ring travels only a short distance from the prong’s exit before striking the nasal vestibule wall (within ~0.06 s of the pulse onset). Importantly, these vortex impingement events coincide with localized pressure maxima and rapid pressure transients, underscoring that their primary relevance in the present context is pressure modulation rather than detailed vortex topology. While the left and right nasal cavities exhibit different flow patterns due to the septal deviation, the overall pressure modulation mechanism (pulsatile jet impinging and raising pharyngeal pressure) occurs in both sides and underlies our main conclusions. Separate per-cavity analysis was therefore not needed for the scope of this study.

In an idealized setting (e.g., a vortex ring impinging on a flat or inclined plate), one would expect the ring to induce a wall boundary layer and possibly form a secondary vortex ring from the shed vorticity. Indeed, previous experiments have documented that when a vortex ring impacts a surface at normal incidence, a sheet of opposite-sign vorticity is generated on the wall, which rolls up into a secondary ring, causing the primary ring to slow and “rebound” away from the wall.^14^ In our anatomical scenario, the same fundamental process likely begins (the pulse-generated vortex creates a region of wall vorticity), but the outcome diverges due to geometric complexity. The confined nasal cavity does not provide the flat, open surface needed for a symmetric secondary vortex ring to fully develop. Instead, the primary vortex’s collision with the uneven nasal walls almost immediately disrupts its coherent structure. This is analogous to the high-confinement cases reported by Ahmed and Erath^13^, where the vortex ring’s interaction with a cavity lip generated intense vorticity that halted the classical secondary ring formation. In our simulations, we did not observe a clear secondary vortex ring separating and orbiting the primary ring. This is likely because any nascent secondary vortex is quickly absorbed into the complex, turbulent flow that ensues in the nasal passage. Notably, the vortex did not exhibit a pronounced rebound as seen in simpler vortex–wall studies, which again can be attributed to the anatomical channel “capturing” the vortex rather than allowing it to ricochet. These differences highlight how anatomical confinement alters the dynamics of the vortex ring: the upper airway’s irregular geometry essentially short-circuits the neat sequence of secondary and tertiary ring formation and rebound that occurs for rings impinging on simpler surfaces.

Despite the altered progression, our findings remain consistent with the physics of vortex–boundary interactions as described in prior studies. For instance, the vortex impingement in the nasal cavity produced transient high pressures at the contact region (Fig. 6d), consistent with the notion that a vortex ring approaching a wall causes an initial stagnation pressure rise at the impact point. Furthermore, the flow immediately after impingement became highly three-dimensional and asymmetric, as the left and right nasal passages exhibited different vortex behavior (one side experienced a stronger jet impact and higher wall shear, as discussed below). This observation aligns with findings from inclined-wall collisions of vortex rings, where one side of the ring interacts more strongly, resulting in unevenly distributed vorticity. *Lim (1989)* documented, via dye visualization, that an inclined impact causes the near-wall side of the ring to form helical vortex filaments that convect away from the wall, eventually ejecting fluid radially in the symmetry plane.^15^ In our case, the patient-specific anatomy inherently creates an “inclined” or uneven impingement – for example, the left nasal passage had a sharper turn and narrower channel, leading to a more forceful vortex impact on the left lateral wall. As a result, we observed significantly higher wall shear stress on the left side of the nasal vestibule compared to the right (Fig. 9a vs 9b), an asymmetry directly attributable to the flow–structure interplay. This is qualitatively similar to prior vortex ring experiments at oblique angles, which found that the portion of the ring hitting first produces more intense local vorticity and a decidedly asymmetric flow pattern. Thus, even though the anatomical airway yields a very complex flow, the underlying phenomena of vortex generation, wall vorticity shedding, and asymmetric vortex stretching are grounded in classical vortex dynamics. Our study illustrates how classical vortex dynamics are modified by anatomical confinement, providing a bridge between fundamental vortex ring physics and the behavior of pulsed jets in the human upper airway.

### Pressure oscillations and OSA therapeutic implications

A key finding of this work is that the beneficial pressure oscillations produced by the pulsatile flow are largely confined to the upper airway (nasal cavity and pharynx). Importantly, the therapeutic relevance of pulsatile flow in this context lies in its ability to increase time-averaged pharyngeal pressure, consistent with established OSA treatment principles, rather than in the oscillatory pressure amplitude itself. The frequency analysis (Fig. 10) shows that the imposed 20 Hz pulsation is strong in the nasal region, but its amplitude decays progressively as the flow moves downstream; by the time airflow reaches the larynx and trachea, the 20 Hz component is almost entirely dissipated into broadband turbulence. In other words, the pulsatile jet delivers oscillatory pressure energy to the nose and throat, but very little of that organized oscillation penetrates the lower airway. This outcome is highly desirable for treating OSA. The pharyngeal airway is the segment that requires stenting pressure to prevent collapse, whereas the lungs and distal airways do not benefit from (and could be disturbed by) large alveolar pressure swings. By confining the oscillatory pressures to the upper airway, the pulsatile airflow targets the therapeutic effect where it is needed (to splint the collapsible airway) without subjecting the lower airways to strong pressure fluctuations. This is in contrast to high-frequency oscillatory ventilation, for example, which intentionally transmits oscillations to the lungs for gas exchange. In our application, we specifically *do not* want to oscillate the alveoli. Our LES results suggest that the complex nasopharyngeal geometry acts as a filter, rapidly absorbing and dispersing the pulsation energy. As the jet’s vortical structures interact with the narrow passages and sharp bend into the oropharynx, vortex breakdown and broadband turbulence emerge, c.f. Fig. 10. This effectively damps the 20 Hz component beyond the pharynx. The behavior is consistent with established vortex dynamics, where coherent vortex rings lose coherence and dissipate their energy upon interacting with boundaries.^13,24^ Here, the *upper airway serves as a boundary-rich environment* that dissipates pulsatile energy before it can reach the lungs. Clinically, this means a pulsating nasal airflow device could provide oscillating positive pressure to splint the pharyngeal region (where OSA collapse occurs) while delivering a relatively steady flow to the lungs.

Another important observation is that the mean pressure elevations achieved with pulsatile flow were higher in the pharyngeal region than those from an equivalent steady flow (i.e., HFNC). Our simulations showed that, for the same mean flow rate, the time-averaged pressure in the pharynx was up to 50% higher with the 20 Hz pulsed jet than with continuous flow (Fig. 8). This corroborates recent patient measurements by Oren et al., who reported that pulsating airflow via nasal cannula attained pharyngeal pressures up to 20 cmH₂O, significantly exceeding what a continuous high-flow could produce. The mechanism for this pressure boost in our model is tied to the transient jet dynamics: during each pulse “on” phase, a surge of flow impinges on the airway walls, locally augmenting the pressure, and these surges occur repeatedly at 20 Hz. The pharyngeal airway, being a collapsible tube, benefits from even a brief high-pressure pulse because it resists collapse at that moment. The pulsatile nature effectively supplies a series of mini-CPAP breaths each second. Meanwhile, between pulses, pressure dips closer to baseline; however, the airway does not immediately collapse in these brief intervals, and the next pulse arrives to reinforce the stent.

We note that the boundary condition formulation used here prescribes inspiratory flow at the nares and atmospheric pressure at the tracheal outlet. As a result, the total flow reaching the trachea differs between the no-therapy baseline (30 L/min), continuous HFNC (effectively 70 L/min total), and the pulsatile case (which varies in time between 30 and ~150 L/min at the nares). If instead a constant tracheal flow rate of 30 L/min were prescribed across all cases, the intraluminal pressure distribution and vortex dynamics in the upper airway would likely differ, because the nasal cavity flow field would adjust to accommodate both the inspiratory demand and the injected flow within a constrained downstream flow rate. While the fluid-mechanical insights into vortex formation, wall impingement, and spectral attenuation would remain qualitatively applicable, the quantitative comparison of therapeutic pressure levels across conditions would be affected. Future studies that prescribe physiologically matched tracheal flow rates or couple the upper airway model with a lung impedance model would strengthen the clinical interpretation of pulsatile versus continuous delivery.

Our discussion thus far suggests that pulsating jet airflow marries two beneficial aspects: (1) a higher peak pharyngeal pressure (for better splinting) and (2) localization of pressure oscillations to the upper airway (to avoid unnecessary lung stress). These features provide pulsatile nasal airflow with a promising clinical profile for OSA therapy, potentially addressing some limitations of both CPAP (the need for a sealed interface to pressurize the airway) and HFNC (insufficient pressure support). Taken together, these findings offer a mechanistic foundation for designing next-generation OSA treatments that deliver positive airway pressure without a mask.

The present simulations are intentionally limited to the inspiratory phase to isolate jet formation, vortex evolution, and pressure-generation mechanisms in the upper airway; extending them to the expiratory phases, which involve different physiological and mechanical considerations, is an important topic for future work.

### Advancing understanding of jet–wall and vortex–structure interactions

Building on the vortex evolution and asymmetry described in Section "Comparison to prior vortex ring studies", this study provides broader insight into jet–wall and vortex–structure interactions in an anatomically realistic geometry. Previous vortex ring experiments have typically been conducted in simplified configurations (straight nozzles, flat or smoothly curved targets, or inclined surfaces) to isolate canonical flow behaviors. In contrast, the upper airway presents a series of angled surfaces, curvatures, and bifurcating passages that fundamentally alter vortex development and breakdown.

Our results demonstrate how classical vortex–wall interaction phenomena adapt in this setting. The pulse-generated vortex initially exhibits behavior consistent with prior studies, forming near the prong, increasing in diameter, and slowing as it moves into the wider nasal cavity, indicative of vortex stretching. However, this coherence is short-lived. Almost immediately, the vortex encounters geometric confinement between the septum and lateral wall, altering its trajectory and structure. The simulation captures early stages of secondary vorticity development at wall impact (Fig. 6d), but this vorticity is rapidly absorbed into the surrounding turbulent flow. From an engineering perspective, this indicates that jet–wall interaction in a tortuous geometry is a highly efficient vortex “breaker,” rapidly converting coherent structures into smaller-scale turbulence through repeated anatomical interactions.

Importantly, the patient-specific anatomy introduces pronounced asymmetry in these interactions. Differences between the left and right nasal passages lead to markedly different flow splits and wall impacts, with the left passage functioning similarly to a more inclined or constricted impingement scenario, producing higher shear and a more energetic vortex impact. This behavior underscores how individual anatomy can strongly modulate jet delivery and vortex evolution, a factor not captured in idealized experimental configurations and one that should be considered in the design of pulsatile airflow therapies.

Finally, the study contributes to understanding jet–wall interactions under pulsatile conditions. Prior research have shown that pulsating jets can enhance surface interactions, for example, increasing heat transfer or erosion by repeated vortex impingement.^25–27^ In our simulations, pulsation similarly amplifies wall interaction in the nasal cavity. During the pulse-on phase, WSS (Fig. 9) was much higher than under steady flow, indicating more vigorous wall jets and recirculation induced by the vortex rings. While excessive shear stress may raise concerns about nasal comfort or mucosal health, the peak shear stress levels remained localized and confined to small regions associated with anatomical constrictions. Interestingly, these elevated shear stress zones could offer secondary benefits, such as promoting mucociliary clearance or reducing stagnant zones in the nasal passages, analogous to how oscillatory airflow is sometimes used in chest physiotherapy to mobilize secretions.^28^ This remains speculative, but it highlights the multifaceted nature of pulsatile jet flows. The present simulations, focused on flow physics, lay the groundwork for future studies to explore such bioengineering aspects.

## Conclusion

In summary, this work has demonstrated that pulsating nasal airflow generates complex vortex-driven flows that effectively elevate pharyngeal pressure while confining oscillatory energy to the upper airway. The results compare favorably with prior vortex ring studies, extending the knowledge of classical vortex-wall interactions into a biomedical setting. From a clinical biomechanics standpoint, the introduction of vortex pulses into the nasal airway emerges as a powerful mechanism to stabilize the collapsible pharynx in OSA. From an engineering standpoint, the study advances our understanding of how pulsed jets behave in non-ideal, convoluted domains – a step beyond canonical fluid mechanics problems. We emphasize that our simulations cover only the inspiratory phase; potential effects during expiration (such as those relevant in obstructive lung disease) were beyond our scope. Additionally, the use of a single OSA patient limits generalizability, as the flow patterns observed here are shaped by this subject’s specific septal deviation and airway asymmetry. Future investigations will be needed to examine different pulse frequencies, waveforms, wall compliance, and extension to multiple anatomies spanning a range of OSA severity before population-level conclusions can be drawn. Nevertheless, the insights gained here suggest that leveraging vortex dynamics in the upper airway could offer a compelling new avenue for mask-free airway pressurization in OSA therapy. By marrying the clinical and engineering perspectives, we gain confidence that (vortical) pulsatile airflow can be tuned to maximize upper airway pressure benefits, minimize adverse effects, and ultimately improve therapy for OSA and related conditions.

## Data Availability

Findings of this study are available from the corresponding author upon reasonable request.
